# The Molecular Chaperone CCT/TRiC: An Essential Component of Proteostasis and a Potential Modulator of Protein Aggregation

**DOI:** 10.3389/fgene.2020.00172

**Published:** 2020-03-19

**Authors:** Julie Grantham

**Affiliations:** Department of Chemistry and Molecular Biology, University of Gothenburg, Gothenburg, Sweden

**Keywords:** molecular chaperone, proteostasis, chaperonin, aggregation, CCT, TRiC

## Abstract

Chaperonin containing tailless complex polypeptide 1 (CCT) or tailless complex polypeptide 1 ring complex (TRiC) is an essential eukaryotic molecular chaperone. It is a multi-subunit oligomer of two rings of eight individual protein subunits. When assembled, each of the eight CCT subunits occupies a specific position within each chaperonin ring. Thus a geometrically defined binding interface is formed from the divergent sequences within the CCT subunit substrate binding domains. CCT is required for the folding of the abundant cytoskeletal proteins actin and tubulin, which in turn form assemblies of microfilaments and microtubules. CCT is also involved in the folding of some additional protein substrates and some CCT subunits have been shown to have functions when monomeric. Since observations were made in worms over a decade ago using an RNAi screen, which connected CCT subunits to the aggregation of polyglutamine tracts, a role for CCT as a potential modulator of protein aggregation has started to emerge. Here there will be a focus on how mechanistically CCT may be able to achieve this and if this potential function of CCT provides any insights and directions for developing future treatments for protein aggregation driven neurodegenerative diseases generally, many of which are associated with aging.

## Introduction

The maintenance of proteostasis is paramount to cellular health. Numerous events contribute to proteostasis, such as transcription/translation and proteolysis, to ensure that protein levels are optimal. In between this “birth and death” of proteins, the molecular chaperones provide assistance to many proteins, ensuring correct conformations are reached/maintained and misfolded proteins can be unfolded to either refold or be degraded. The array of chaperones present in the cell, the “chaperome,” can change during aging and in neurodegenerative disease states and a detailed analysis of this, performed by Brehme et al. (2014), demonstrates that during aging there is a trend where ATP-dependent chaperones are down regulated, whilst ATP-independent chaperones are up regulated. These observations indicate potential complex changes in the chaperoning requirements and chaperoning capacities of cells. If cells become deficient in their chaperoning capacity during aging or if proteostasis is disrupted as a consequence of protein misfolding diseases, then modulation of chaperone activity may provide a mechanism to combat the formation of potentially toxic protein aggregates. The chaperones consist of several different families of proteins, which contribute to maintaining proteostasis by different mechanisms. For example, the Hsp70 family, together with the co-chaperone Hsp40 act by binding to nascent or unfolded polypeptide chains via stretches of hydrophobic sequences, whilst Hsp90 proteins bind to and stabilize a more limited sub set of proteins, both in an ATP-dependent manner. Conversely, the small Hsp proteins prevent aggregation via ATP-independent mechanisms (for a comprehensive review, see for example, Hartl et al., 2011). The focus of this review is the ATP-dependent chaperone chaperonin containing tailless complex polypeptide 1 (CCT) also known as tailless complex polypeptide 1 ring complex (TRiC) and a potential role for CCT in the modulation and/or suppression of protein aggregation will be discussed.

## The Molecular Chaperone CCT

**FIGURE 1:**
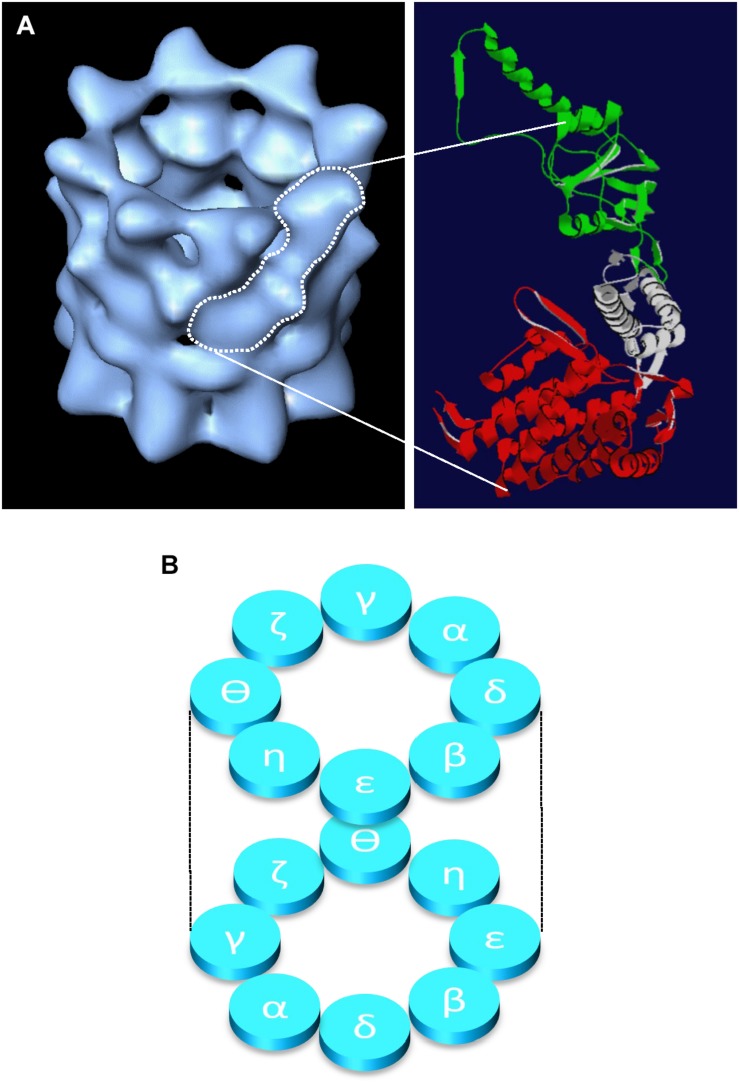
Structure of the CCT oligomer. The structure of the CCT oligomer obtained by cryo-electron microscopy and three-dimensional reconstruction (Llorca et al., 2001) is shown in **(A)**, left hand side with the approximate density of one CCT subunit outlined in white. The domain architecture of a single CCT subunit is shown in **(A)**, right hand side based on the crystal structure of the alpha subunit of the thermosome (PDB 1A6D), where the equatorial domain (containing the nucleotide binding pocket) is shown in red, the flexible linker domain in gray, and the apical substrate binding domain is in green (adapted from Vallin and Grantham, 2019). The inter- and intra-ring subunit arrangements (Leitner et al., 2012; Kalisman et al., 2012) are shown in panel **(B)**.

CCT is a member of the chaperonin family of molecular chaperones and is found in all eukaryotes. Eight individual protein subunits, named either α to θ or 1 to 8 (all products of essential genes in yeast) assemble to form a double-ringed barrel structure (Figure 1), which is required for the folding of newly synthesized actin and tubulin molecules and a somewhat restricted range of other folding substrates. Whilst the number of proteins that require CCT for folding is often a matter for debate, the cytoskeletal proteins actin and tubulin are highly abundant proteins and thus account for the majority of substrate proteins found bound to the CCT oligomer at any one time (Grantham et al., 2006; reviewed by Willison, 2018; Vallin and Grantham, 2019). Actin and tubulin are considered obligate folding substrates as they are dependent upon CCT to fold, whilst some substrates may be more opportunistic interaction partners, which gain assistance from CCT if off-pathway folding occurs, but are not usually dependent upon interactions with CCT for their folding.

In addition to the folding of newly synthesized proteins by the CCT oligomer, CCT oligomer function also extends to assisting in the assembly of complexes between the Von Hippel Lindau tumor suppressor protein and elongin proteins (Melville et al., 2003) and CCT also binds to the actin filament capping and severing protein gelsolin (Brackley and Grantham, 2011). In the case of gelsolin: CCT interactions, gelsolin appears not to be folded by CCT (Brackley and Grantham, 2011), but instead CCT may have a role in modulating the activity of gelsolin (Svanstrom and Grantham, 2016). Furthermore, CCT subunits when monomeric may also possess functions independent of protein folding. For example, CCT5(ε) may act as a component of the SRF signaling pathway via its ability to bind to the co-transcriptional activator MRTF-A (Elliott et al., 2015), whilst CCT4(δ) localizes close to the plasma membrane and is a binding partner of the dynactin complex protein p150^Glued^ (Spiess et al., 2015; Echbarthi et al., 2018). Thus, CCT activity extends beyond the folding of newly synthesized proteins and in addition to the functions performed by the CCT oligomer, CCT subunits in their monomeric forms can have distinct functions.

## Inhibition/Modulation of Protein Aggregation Via CCT

Nollen et al. (2004) first identified CCT as a potential modulator of protein aggregation by performing an RNAi screen in *Caenorhabditis elegans* to detect which genes would result in the early aggregation of polyglutamine tracts when gene expression was reduced. In this screen, six CCT subunits, Hsp70 and DnaJ were identified within the “protein folding” functional category of such genes and the authors comment that the range of chaperone proteins identified was somewhat limited (Nollen et al., 2004). Consistently, in *C. elegans*, reduction of CCT subunits via RNAi resulted in a reduction in mobility in early onset paralysis assays when expressing Aβ_42_ and polyglutamine Q35 as model aggregating proteins but not in wild type animals, whilst in HeLa cells expressing Htt-exon1(Q78)-GFP, reduction of all except CCT5(ε) leads to increased aggregate formation (Brehme et al., 2014).

It is not surprising that the reduction in the levels of certain chaperone proteins could exacerbate the aggregation of a vulnerable polyglutamine tract. Whilst Hsp70 and DnaJ are considered to be more general chaperones, CCT has a rather more restricted range of folding substrates. Therefore, the question arises of whether the polyglutamine tract itself has requirements upon CCT for folding or stabilization or if the effect is indirect. The latter situation would be the case if the depletion of CCT resulted in the misfolding of a CCT folding substrate and the subsequent loss of function or toxic gain of function affected the polyglutamine tract-containing protein. It is therefore interesting to note that the β-tubulin isoform tbb-2 is also detected in the *C. elegans* screen as tubulin is a folding substrate of CCT (Nollen et al., 2004).

Although the information gained from such screens can be interpreted in different ways, a direct role for CCT in modulation of polyglutamine tracts has now been shown. In *in vitro* reconstitution experiments performed with an aggregating form of huntingtin exon 1, Htt53Q, Behrends et al. (2006) showed that CCT oligomer alone was able to reduce the formation of large, insoluble aggregates in both the presence and absence of nucleotide suggesting a rather passive mechanism. However, CCT together with Hsp70 promoted the formation of soluble Htt53Q oligomers of approximately 500 kDa (Behrends et al., 2006). Consistently, Tam et al. (2006) showed that the CCT oligomer can inhibit the formation of huntingtin aggregates and furthermore demonstrated that in yeast, the over-expression of single CCT subunits can, in the case of CCT1(α) and CCT4(δ), change the morphology of huntingtin aggregates. The substrate-binding apical domain (see Figure 1) of CCT1(α) alone was sufficient to suppress the aggregation *in vitro* of Q51 upon its cleavage from glutathione S-transferase (Tam et al., 2006). These observations raise intriguing questions about the mechanisms that facilitate this aggregation-suppression behavior of CCT, as an apical domain alone cannot give mechanical input driven by the binding/hydrolysis of ATP. However, the CCT1(α) apical domain was later identified as a site important for binding to a helix located toward the N-terminus of huntingtin that can act as a switch for initiating aggregation and thus CCT binding to this site can hinder aggregation (Tam et al., 2009).

The potential of CCT1(α) to be therapeutically active was examined by Sontag et al. (2013) where yeast CCT1(α) apical domain was applied to PC12 cells and striatal cells derived from a mouse knock in both expressing aggregating model versions of the huntingtin protein. It was found that the CCT1(α) apical domain was able to reduce inclusion body formation in PC12 cells and also increased respiration rates within the striatal cells, implying a reduction in the toxicity arising from expressing the huntingtin variant (Sontag et al., 2013). In addition to showing a possible therapeutic mode of action by CCT1(α) apical domain, a second important observation was made with regard to the ability of CCT1(α) apical domain to pass through the plasma membrane of the PC12 cells to enter cells (Sontag et al., 2013). This translocation may be mediated via a sequence within CCT1(α) apical domain that is similar to HIV trans-activator of transcription (Sontag et al., 2013).

## Potential Mechanisms of Action

Are the effects of CCT on suppression of polyglutamine tracts specific or a result of a more general mechanism? CCT is not unique in its ability to modulate this aggregation and as already discussed, the Hsp70 chaperone system can exert effects. It is also interesting to note that the prefoldin chaperone, a multi-subunit co-chaperone, which interacts with folding intermediates upstream of CCT (reviewed by Grantham, 2010) may also modulate the aggregation of the huntingtin protein (Tashiro et al., 2013). As already discussed above, CCT appears to be able to modulate the aggregation of the huntingtin polyglutamine tract when both oligomeric and in the case of CCT1(α), as a monomer. These observations raise intriguing questions regarding potential mechanisms of action. Tomography analysis suggests that the CCT oligomer can cap fibrils formed from aggregating polyglutamine tracts, whilst cryo-electron microscopy suggests that small polyglutamine oligomers may be sequestered within the cavity of the CCT oligomer (Shahmoradian et al., 2013). A homo-oligomer formed from the CCT5(ε) subunit also appears to cap mutant huntingtin fibrils (Darrow et al., 2015), suggesting even non-physiological assemblies of CCT can possess a fibril capping activity.

In addition to the aggregation of the huntingtin protein being modulated by CCT, CCT oligomer can also prevent the Parkinson’s disease-causing A53T mutant of α-synuclein and wild-type α-synuclein from aggregating in the presence of either ATP or ADP (Sot et al., 2017). Similar to the observations made with the huntingtin protein by Shahmoradian et al. (2013), α-synuclein oligomers also interact with CCT and CCT is able to diminish toxicity in the neuroblastoma cell line SH-SY5Y (Sot et al., 2017). Both in the case of huntingtin (Behrends et al., 2006) and α-synuclein (Sot et al., 2017) effects upon aggregation are seen regardless of ATP or ADP being present, suggesting a passive mechanism.

The fibril-capping activity displayed by the CCT oligomer may indicate a distinct ability for the capping of growing protein fibers by CCT, where changes in conformation will likely be occurring within the protein as it polymerizes. Indeed it has been demonstrated that *in vitro*, CCT oligomer is able to reduce the initial rate of polymerization of actin filaments, probably by acting at the fast-growing filament plus-ends (Grantham et al., 2002). Interestingly the bacterial chaperonin GroEL was shown to have no effect on actin polymerization (Grantham et al., 2002) and little effect on α-synuclein fibrillation (Sot et al., 2017), indicating a degree of chaperonin specificity.

**FIGURE 2:**
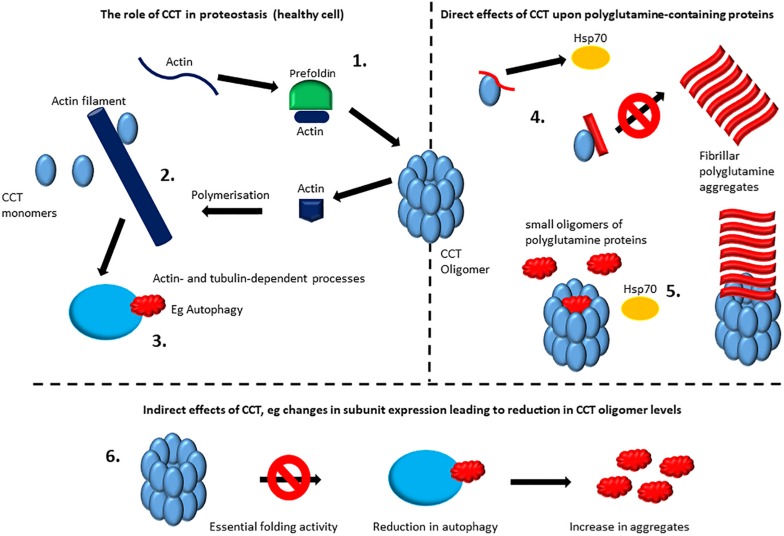
Direct and indirect effects of CCT upon protein aggregation. The role of CCT in proteostasis: the CCT oligomer is involved in the folding of numerous proteins including actin and tubulin which require interactions with CCT and for some proteins this will involve interactions with the chaperone prefoldin before binding to CCT **(1)**. For actin filaments and microtubules there is an extended role for CCT including some interactions with monomeric CCT subunits **(2)** (reviewed by Vallin and Grantham, 2019). Actin filaments and microtubules systems will then have an impact upon proteostasis. For example, a functional actin network is required for autophagy **(3)** (Pavel et al., 2016), whilst microtubule-mediated transport is also important for distribution and fate of polyglutamine aggregates (Webb et al., 2004). For ease of illustration only actin filaments are depicted here. Direct effects of CCT upon polyglutamine containing proteins: some CCT subunits when monomeric may reduce aggregation/modulate morphology (Tam et al., 2006) possibly by providing a specific interaction surface for stabilization before irretrievable aggregates form, buying time for other chaperones such as the Hsp70/Hsp40 system to resolve the misfolded proteins. In the case of the CCT1(α) apical domain a more specific mechanism may be involved via binding to a helix located toward the N-terminus of huntingtin that can act as a switch for initiating aggregation and thus CCT binding to this site can hinder aggregation (Tam et al., 2009) **(4)**. The CCT oligomer can directly cap fibrils formed from aggregating polyglutamine tracts, whilst small polyglutamine oligomers may be sequestered within the cavity of the CCT oligomer (Shahmoradian et al., 2013) and may reduce aggregate formation or (together with Hsp70) promote the formation of soluble Htt53Q oligomers of approximately 500 kDa (Behrends et al., 2006) **(5)**. Indirect effects of CCT: Reduction in expression levels during aging or disease, such as those documented by Brehme et al. (2014) could lead to reduced levels of function CCT oligomer, which could result in failures in pathways mediated by proteins that are dependent upon, for example, actin and tubulin. For ease of illustration only autophagy is depicted here **(6)**.

However, despite growing evidence that CCT can directly modulate polyglutamine tract-containing proteins (as described above), Pavel et al. (2016) demonstrate that the increases in the number of cells containing polyglutamine aggregates seen in several model systems when CCT subunits are depleted by siRNA-targeting, are in fact due to a compromised autophagy pathway. These defects in autophagy arise due to effects upon the actin cytoskeleton when CCT subunits are depleted presumably via compromised actin folding (Pavel et al., 2016). Importantly, depleting CCT subunits in an autophagy-deficient background did not further increase the aggregation of mutant huntingtin or the aggregation of another polyglutamine-containing protein ataxin 3 (Pavel et al., 2016). Furthermore, the CCT oligomer is involved in folding components of the mTORC complex, thus affecting mTORC assembly (Cuellar et al., 2019). Therefore, loss of CCT oligomer could influence several cellular processes including autophagy via mTORC-mediated pathways. A cartoon depicting direct and indirect ways in which CCT could influence proteostasis is shown in Figure 2.

Although many of the effects of reducing CCT levels by siRNA upon the enhanced aggregation of polyglutamine-containing model proteins may well be due to loss of CCT oligomer activity, and thus failure of the autophagy pathway (Pavel et al., 2016), the effects of the CCT oligomer on the modulation of huntingtin aggregates are clear (e.g. Behrends et al., 2006; Tam et al., 2009). Furthermore, the observations that increasing individual CCT subunit levels has beneficial effects are intriguing mechanistically (Tam et al., 2006, 2009; Sontag et al., 2013). A single subunit or apical domain alone will have limited mechanistic potential to modulate the structure of a substrate in comparison to the intact oligomer where force can be generated by multiple substrate binding sites (Llorca et al., 1999; reviewed by Vallin and Grantham, 2019). However, a single subunit or apical domain may well act by providing a specific interaction surface for stabilization before irretrievable aggregates form, buying time for other chaperones such as the Hsp70/Hsp40 system to resolve the misfolded proteins. The involvement of single CCT subunits with other proteostasis components has been shown to occur in the case of CCT3(γ) over-expression reducing HTTQ97 levels in neurons from the BACHD mouse (a mouse line harboring a 97 repeat length of polyglutamine), where functional proteasomes were required (Zhao et al., 2016).

## CCT and Axonal Transport

Neurons often display a highly polarized morphology and are therefore especially dependent upon axonal transport to maintain cellular fitness, as components synthesized in the cell body will be required substantial distances away (reviewed by Millecamps and Julien, 2013). Localized protein synthesis is also known to occur in both dendrites and axons (reviewed by Holt et al., 2019) and thus within axons and dendrites there will be numerous requirements for the proteostasis machinery. Several CCT subunits themselves have been shown to associate with the slow component b during axonal transport in rat sciatic nerve, together with the constitutively expressed molecular chaperone Hsc70 and actin (Bourke et al., 2002). Using a neuron-pairing approach between wild-type mouse neurons and neurons from the BACHD mouse, Zhao et al. (2016) found that both anterograde and retrograde transport of brain-derived neurotrophic factor (BDNF) was negatively affected and that expression of either CCT3(γ) or the apical domain alone of CCT1(α) could contribute to the rescue of this polyglutamine-mediated effect.

The over expression of CCT5(ε) was also shown to increase the retrograde transport of BDNF in wild type neurons (Chen et al., 2018). Interestingly, tau protein was required for this effect and CCT5(ε) appeared to be connected to increased CDK5 activity and subsequent tau phosphorylation/effects on microtubule bundling (Chen et al., 2018). Therefore several CCT subunits, when in their monomeric forms, appear to have the potential to influence axonal transport, revealing a further level of complexity of the functions of the CCT molecular chaperone.

## Changes in CCT Gene Expression in Aging and Disease

In human brain samples, CCT1(α), CCT2(β), CCT4(δ), and CCT6(ζ) appear to be repressed during aging, with repression also seen in Alzheimer’s and Huntington’s disease, albeit with varying significance, whilst in Parkinson’s disease expression level trends are less clear (Brehme et al., 2014). The consequences of changes in CCT subunit expression profiles are potentially complex. Loss of one of the eight CCT subunits in cultured mammalian cells (by siRNA depletion) leads to loss of the CCT oligomer with the non-targeted subunits present as monomers (Grantham et al., 2006; Brackley and Grantham, 2010). Based on the gene expression data presented by Brehme et al. (2014), during aging, Alzheimer’s and Huntington’s disease, one might expect various processes, including autophagy, to be effected by loss of assembled CCT oligomer due to repression of one or more CCT genes.

The mechanisms of altered CCT expression in neuronal cells are unclear. However, a connection between CCT4(δ) and the vaccinia-related kinase 2 (VRK2) has been reported where despite CCT4(δ) not being a substrate for phosphorylation by VRK2, VRK2 was shown to bind to the C-terminal portion of CCT4(δ) (Kim et al., 2014). Over-expression of VRK2 was shown to result in a slight decrease in CCT4(δ) levels via ubiquitin-mediated degradation (Kim et al., 2014).

In addition to changes in the expression of CCT subunits occurring during aging and disease, mutations in CCT4(δ) and CCT5(ε) have been identified which may be associated with hereditary sensory neuropathies (Lee et al., 2003; Bouhouche et al., 2006). In both cases the mutation was in the equatorial domain and would presumably be linked to some degree of compromised CCT activity.

## Discussion

In healthy cells, the oligomeric CCT will play an essential role in proteostasis by mediating the folding of its obligate substrates (including the abundant cytoskeleton components actin and tubulin) and an array of lower abundance proteins. In an additional layer of complexity, some of the CCT subunits have also been shown to have functions in their monomeric states, thus the expression levels of the eight CCT subunits and the assembly state of the chaperonin oligomer will be important factors determining the extent of CCT functions. As changes in CCT subunit expression occur in aging and in several neurodegenerative diseases (Brehme et al., 2014) CCT activity will be altered and the consequences potentially difficult to predict. Furthermore, increased levels of post-translational modifications to aggregating proteins, such as phosphorylation of tau, are observed during neurodegenerative aggregation (Ayyadevara et al., 2016). If these modified forms of proteins are recognized to a lesser degree than the unmodified forms then this would compound the effects of reduced chaperones during aging and neurodegenerative diseases.

Therapies that aim to boost the levels of functional CCT oligomer may be extremely difficult to develop due to the need for the appropriate expression levels of eight individual CCT genes, which are distributed over seven chromosomes. However, the findings that the apical domain of CCT1(α) is sufficient to modulate aggregation and is able to cross the plasma membrane (Sontag et al., 2013) greatly augments the potential of using CCT-mediated therapies for targeting protein misfolding diseases. As drug delivery often poses insurmountable challenges, the utilization of an inherent membrane permeability is highly advantageous. Possible approaches may be to deliver the CCT1(α) apical domain to cells concurrently with therapeutic agents that stimulate a heat shock response to increase levels of (amongst other chaperones) the Hsp70/Hsp40 chaperone system.

Of course modulation of protein aggregation is likely to remain an extremely complex task as modulation of proteostasis capacities may have unpredictable and unfavorable consequences. For example, inhibition of fibril formation may result in increases in the levels of oligomeric intermediate species, which themselves may be more toxic than the fibrils. Thus understanding the functional range of proteostasis components with regard to protein aggregation is the first step in managing age-related neuropathies.

## Author Contributions

JG wrote the review as sole author.

## Conflict of Interest

The author declares that the research was conducted in the absence of any commercial or financial relationships that could be construed as a potential conflict of interest.
